# New Approaches to Identify Sepsis Biomarkers: The Importance of Model and Sample Source for Mass Spectrometry

**DOI:** 10.1155/2020/6681073

**Published:** 2020-12-24

**Authors:** Angélique Blangy-Letheule, Antoine Persello, Bertrand Rozec, Michel De Waard, Benjamin Lauzier

**Affiliations:** ^1^Université de Nantes, CHU Nantes, CNRS, INSERM, l'institut du thorax, F-44000 Nantes, France; ^2^InFlectis BioScience, Nantes, France; ^3^Labex ICST, Valbonne, France

## Abstract

Septic shock is a systemic inflammatory response syndrome associated with circulatory failure leading to organ failure with a 40% mortality rate. Early diagnosis and prognosis of septic shock are necessary for specific and timely treatment. However, no predictive biomarker is available. In recent years, improvements in proteomics-based mass spectrometry have improved the detection of such biomarkers. This approach can be performed on different samples such as tissue or biological fluids. Working directly from human samples is complicated owing to interindividual variability. Indeed, patients are admitted at different stages of disease development and with signs of varying severity from one patient to another. All of these elements interfere with the identification of early, sensitive, and specific septic shock biomarkers. For these reasons, animal models of sepsis, although imperfect, are used to control the kinetics of the development of the pathology and to standardise experimentation, facilitating the identification of potential biomarkers. These elements underline the importance of the choice of animal model used and the sample to be studied during preclinical studies. The aim of this review is to discuss the relevance of different approaches to enable the identification of biomarkers that could indirectly be relevant to the clinical setting.

## 1. Introduction

Sepsis and septic shock are common causes for admission to intensive care units. Sepsis is defined as organ dysfunction resulting from a deregulated host response to infection [1]. In 2017, this pathology affected 48.9 million people worldwide, resulting in the deaths of 11 million patients [2]. Over the last 50 years, studies have demonstrated that myocardial dysfunction is a common finding in septic patients and approximately 50% of septic patients present signs of myocardial decompensation with variable development kinetics depending on the patient resulting in excess mortality of more than 60% [3]. Oxygen delivery is impaired in the tissues of sepsis patients with organ dysfunction. Septic shock is the most severe manifestation of sepsis. It is characterized by persistent hypotension, associated with metabolic dysfunction and significant tissue suffering. A 10% increase in mortality is associated with septic shock for each additional hour of delay between the diagnosis and the implementation of an adequate treatment in hospital settings [4]. Since 2017, the World Health Organization (WHO) has made sepsis one of the world's top priorities and has adopted resolutions to improve the prevention, diagnosis, and management of this disease [5]. Septic shock is a complex and multifactorial pathology presenting a great heterogeneity of clinical manifestations. This therefore explains the differences in the kinetics of organ dysfunction and complicates its early diagnosis and appropriate management. Timely management could save 80% of patients with sepsis [6]. The use of early biomarkers and therapeutic targets that are sensitive and specific to the evolution of the pathology would facilitate rapid diagnosis and therefore early management of patients, limiting organ dysfunctions, particularly cardiac dysfunction, and optimizing patient chances of survival. Over the last few years, numerous studies have proposed new biomarkers (Table 1). It should be noted that it is easier to standardise experimentation and control the kinetics of the evolution of the pathology in animal studies. It is then possible to, respectively, limit both the phenotypic heterogeneity between individuals and identify early versus late biomarkers. Over the past 20 years, a large number of biomarkers and therapeutic targets related to sepsis have been proposed [7]. However, the inherent heterogeneity of the pathology and the absence of similar reference bases in the different studies have made it difficult to confirm the quality and accuracy of these biomarkers for the diagnosis of sepsis [8]. For research teams working on sepsis, it is therefore important to implement improved investigation methods to generate better results and more reliable biomarkers. In recent years, omics technologies have gained momentum and increasing significance with improved splitting techniques and instrument performance. Analytical approaches have made it possible to identify proteins of low abundance in complex samples thereby improving the identification of new biomarkers. Proteomics based on mass spectrometry (MS) enable the use of different samples such as biological tissues or fluids based on patient cohorts or animal models in order to identify biomarkers and/or therapeutic targets. In the context of septic shock, the ideal biomarker should possess the following qualities: (i) be measurable during the early phase of the pathology, (ii) be easy to detect, (iii) be inexpensive, and (iv) be sufficiently sensitive and specific. The choice of the model and the relevance of the biological samples analysed by proteomics are essential for the identification of clinically usable biomarkers. This review seeks to discuss the quality and properties of different models that will enable the potential identification of clinically relevant biomarkers.

## 2. Mass Spectrometry for the Identification of Sepsis Biomarkers

**Figure 1:**
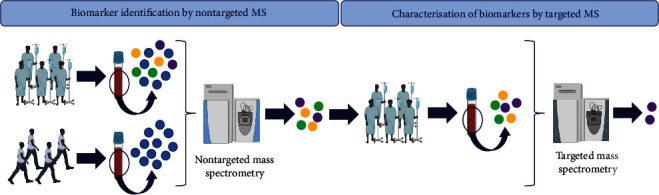
Process implemented in the search for MS-based biomarkers of septic shock. Potential biomarkers are first analysed by nontargeted MS and then characterised by targeted MS.

MS is a very powerful and sensitive analytical method that identifies and quantifies molecules by measuring their mass. MS can provide information on several identified molecules at a time using a targeted approach or hundreds or even thousands of compounds via a nontargeted approach. Nontargeted MS approaches are typically used in the discovery phases to compare samples from two or more different populations. Once a compound that is present at a differential amount between these populations has been identified, a targeted approach can be used at a later stage to characterise the suspected biomarker(s) in a focused manner (Figure 1). Herein, only nontargeted mass spectrometry will be described since it provides identification of a larger set of biomarkers.

In recent years, improvements in selective depletion techniques, splitting techniques, mass spectrometry instrumentation, and analytical approaches have improved proteome analysis. This has led to a better understanding of molecular processes involved in many disease states and to the identification of new biomarkers. An example of the search for diagnostic markers by proteomic analysis is the detection in cerebrospinal fluid of the protein 14-3-3*σ* as a marker of the Creutzfeldt-Jakob disease [19].

### 2.1. MS-Based Proteomics

The study of proteins gained increased maturity in the 1990s with the advent of MS. Over the last ten years, this technique has become an almost indispensable approach for research of diagnostic and prognostic biomarkers as well as for monitoring the development of pathologies. Proteomic studies can be carried out on different samples such as tissues or various biological fluids such as urine or blood. Indeed, they have already been used to identify a panel of metabolites for the stratification of patients suspected of developing sepsis [11]. However, the biological samples selected for identification of biomarkers should be considered carefully. The pathophysiology of sepsis and associated clinical constraints such as accessibility to biological tissue could limit the identification of biomarkers. It is therefore necessary to select the type of sample to be studied during preclinical work to enable identification of biomarkers that are relevant to the clinical setting.

#### 2.1.1. The Search for Biomarkers in Organs

In sepsis, the inflammatory response can lead to damage and failure of organs such as the lungs, heart, or kidneys which are associated with excess mortality. The underlying mechanisms are not well understood, and without adapted diagnostic or prognostic biomarkers, the pathology will evolve toward septic shock and potentially death. In order to search for such biomarkers, several studies have focused on the proteome of organs that were damaged during sepsis itself by performing and studying biopsies. For example, a study on the temporal profile of renal proteome changes induced by sepsis highlighted that ceruloplasmin (CR) and haptoglobin (Hp) are upregulated 90 minutes after the onset of sepsis [20]. Similarly, a cardiac tissue proteome study reported that the oligomerization of pentraxin-3 (PTX-3) increased in patients who did not survive sepsis [21]. The octameric PTX-3 level in patients with sepsis could therefore be predictive of an unfavourable clinical state. In all of these studies, MS, performed on tissue, made it possible to identify proteins that are deregulated during sepsis. These proteins could serve as biomarkers or therapeutic targets specific to a tissue/organ during sepsis. However, potential biomarkers identified by MS must be clinically usable to assist in medical decision-making. Indeed, the study of a tissue requires a biopsy, an invasive procedure, therefore limiting its use for routine biomarker research in clinics (Table 2). As a result, research has shifted toward the study of biological fluids that are more easily accessible in a clinical setting, and in particular, routinely used in clinics for other pathologies [22].

#### 2.1.2. The Search for Biomarkers in Exosomes

Exosomes are membrane vesicles found in many biological fluids (such as blood or urine) that transmit signals between cells [23]. In the urine, the number of exosomes and extracellular vesicles increases continuously between 6 and 48 hours after induction of sepsis, suggesting that they could be potentially involved in this pathology [24]. It has been shown that exosomes play a role in sepsis through the interaction of various compounds released by the septic condition on membrane receptors. Proteomic analyses of exosomes in patient plasma samples could be an effective approach for the identification of protein biomarkers to be used for the diagnosis of sepsis. A study of exosomes in plasma from patients with sepsis identified 238 proteins [25]. Among these proteins, a negative correlation between serine palmitoyltransferase 3 (SPTLC 3) and the progression profile of the pathology was demonstrated, suggesting that SPTLC 3 could play a role in the development of sepsis [23]. SPTLC 3 enables the synthesis of ceramide from palmitate and serine. Studies have shown that sepsis leads to an increase in ceramide levels which play a role in sepsis-induced cardiac dysfunction [25]. Thus, the increase in SPTLC 3 could predict cardiac dysfunction in patients with sepsis. However, it should be noted that the preparation of exosomes is a cumbersome process. The search for biomarkers from exosomes is therefore not the best strategy in the case of sepsis since diagnosis cannot be made rapidly enough. Rapid diagnosis is a key criterion in this pathology to limit the mortality associated with the disease (Table 2).

Most studies to detect biomarkers have focused on the proteome which is defined as set of proteins in a cell compartment, cell, or tissue. More recently, much work has focused on proteins in plasma, serum, or urine. It would therefore be more appropriate in this case to use the term “secretome” which is defined as the set of proteins secreted or liberated by a cell, tissue, or organism at a given time and under given conditions, which would explain their presence in biological fluids [26].

### 2.2. “Secretomics”

Secretome is a dynamic and complex entity that varies according to cell type or organism, functional state, and time. Indeed, depending on the stimuli they receive, the proteins released by a given cell may vary. For example, during an infection, the high-mobility group box 1 (HMGB1), which belongs to the alarmin family, is released into the extracellular space and participates in the pathogenesis of sepsis [10]. Thus, any alteration in the release of a given protein and the abundance of such a protein in a given environment could reflect a pathological state [27]. Although the term secretome was first mentioned in 2000 in a study by Tjalsma et al. on the proteins secreted by the bacterium *Bacillus subtilis*, the concept of circulating factors in plasma/serum is older [28]. Parillo et al. showed 35 years ago that the transfer of serum from patients in septic shock to rat healthy cardiomyocytes induces a decrease in both the extent and velocity of shortening during contraction. This work demonstrated the existence of circulating blood factors favouring myocardial depression during septic shock in humans [29]. More recently, Mastronardi et al. studied this concept and reported that intravenous administration of “microparticles” present in the plasma of patients in septic shock at an early stage leads to increased expression of proinflammatory proteins such as nuclear factor-*κ*B (NF-*κ*B) in the heart and lungs [30]. In the same year, a study by van Hees et al. demonstrated that the transfer of plasma from patients in septic shock to skeletal muscle tissue leads to a loss of myosin from skeletal myocytes. The factors that contribute to such muscle weakness are released during sepsis. Proinflammatory cytokines such as IL-6, TNF-*α*, interferon-*γ*, or interleukin-1*β* (IL-1*β*) are known to be involved in muscle degeneration pathologies [31]. In this study, they showed that the plasma level of IL-6 correlates with the severity of myosin loss. However, it was also found that the addition of IL-6 alone to control plasma is not associated with muscle atrophy [31]. Hence, these results suggest that additional circulating factors in addition to IL-6, not currently identified, are also involved in the transmission and/or amplification of the pathological phenotype according to mechanisms that remain to be defined. The study of proteins that constitute the secretome could lead to a better understanding of the mechanisms underlying septic shock in addition to facilitating the identification of a number of biomarkers or a combination of them (coming as a signature) in the early phase of this pathology. However, analysis of the secretome is rendered difficult by the dynamic range of protein expression which is a major technical difficulty in proteomic studies. For example, plasma contains a wide dynamic range of more than ten orders of magnitude. As a result, 90% of the proteins contained in the plasma consist of only 10 well-identified proteins such as albumin, immunoglobulin, or transferrin [11]. This wide dynamic range of concentrations makes it particularly difficult to analyse proteins of low abundance by MS, thereby hindering the identification of new biomarker candidates. To avoid this, strategies for selective depletion of abundant proteins have been developed to facilitate analyses of the secretome and identify new biomarkers from biological fluids.

#### 2.2.1. Selective Depletion Techniques

Various approaches for processing proteomic samples have been developed in recent years to reduce the complexity of biological samples, including selective depletion. During proteomic analyses, these techniques enable detection of the signal of low abundance proteins in a complex protein sample by reducing the dynamic concentration range of the proteins. Two approaches are particularly used: immunodepletion and ProteoMiner™. The immunodepletion technique is simply based on a pulldown thanks to the interaction between an antibody and a protein in the sample. The choice of antibody or antibodies is based on knowledge of the proteins studied [32]. The immunodepletion process can easily be performed on plasma or serum samples. However, immunodepletion can cause protein-protein interactions, resulting in depletion of nontargeted proteins [33]. In addition, the high cost of sample preparation prevents this method from being routinely used in clinical settings. Contrary to immunodepletion, ProteoMiner™ does not use antibodies. It is based on the interaction of a high combination of 6-amino acid peptide sequences, called hexapeptides, with the proteins from the sample. These sequences are randomly generated so that all of the proteins in the sample will be able to interact with one or more hexapeptides. Since the binding capacity of hexapeptides is limited, a significant fraction of the highly abundant proteins is eliminated during the wash phase. The proteins bound to the hexapeptides will then be recovered during the elution phase for MS analyses *[*34*]*. This technique therefore appears to be particularly appropriate for nontargeted analysis. The biggest inconvenience of using ProteoMiner™ is most probably that the probability of catching small molecular weight proteins or peptides is much lower than larger molecular weight proteins, implying that it has a cut-off efficacy for lower molecular weight proteins (approximately 2 kDa). Hence, the technique is not as good for analysing the relative abundance of cytokines despite their being relevant targets in septic shock.

#### 2.2.2. Detection of Biomarkers in Urine

Since biopsies are not appropriate for the detection of early biomarkers in clinical investigations for sepsis, several studies have turned to urine analyses. The collection of urine samples is noninvasive, unlike blood samples.

The search for early biomarkers in human urine samples has identified 39 deregulated proteins in urine from septic patients. Among these proteins, levels of *β*-2-microglobulin (B2M) and *α*-1-antitrypsin (SERPINA1) are increased during sepsis-induced acute kidney injuries (AKI) while levels of *α* fibrinogen (FGA) chains are decreased. The combination of these markers could therefore predict the onset of AKI [35]. In a recent study, 123 deregulated proteins were detected in urine samples from rats in sepsis or sepsis patients. Among these targets, the acidic nucleic protein deglycase DJ-1 (PARK7) and cadherin 16 (CDH16) were found in samples from both models. This study also showed that the diagnostic sensitivities and specificities of PARK7 and CDH16 were greater than that of neutrophil gelatinase-associated lipocalin (NGAL), which is currently used to diagnose AKI [24]. Thus, these two proteins could potentially be considered as early biomarkers of sepsis-induced AKI. Study of the urinary proteome provides identification of potential biomarkers or therapeutic targets of sepsis. However, patients with sepsis have low diuresis, making it difficult to study urinary biomarkers in the clinic (Table 2). Moreover, a large portion of the deregulated proteins could result from kidney damage, suggesting that they could represent late biomarkers instead of early ones. Therefore, the use of blood samples appears to be more appropriate to look for biomarkers allowing for rapid diagnosis and patient follow-up.

#### 2.2.3. Detection of Biomarkers in Blood Samples

Whole blood consists of plasma, cells, erythrocytes, and platelets. Cells contained in the blood are eliminated to obtain plasma and serum which makes their analyses simpler. As a result, MS studies are mainly performed on plasma or serum samples. Therefore, this review will not address the search for biomarkers in whole blood.

*(1) Serum*. Unlike plasma, serum is devoid of blood cells or fibrinogen. This biological fluid is rich in proteins, easily accessible, and capable of providing dynamic information on the circulatory system and the evolution of the disease. Proteomic analyses of patient sera revealed a combination of ten proteins that are deregulated during sepsis, including antithrombin III (AT-III), clusterin (CLUS), and serum amyloid A-1 (SAA-1) [14]. The latter is increased in the sepsis patient group, indicating a response to inflammation and tissue damage [14]. Similarly, Hayashi et al., who studied the patient proteome over time, showed a significant decrease in haemoglobin beta 1 and 2 chains in the group of patients who did not survive sepsis [36]. These molecules could be markers of sepsis severity. Both studies identified the biomarkers of sepsis in serum. However, there are no common biomarkers between these studies. This can in part be explained by the fact that Hayashi et al. incorporated the concept of kinetics of pathology development into their study which forces one to study the evolution of the secretome and not its state at a given time. Serum samples contain little or no coagulation-associated proteins, but sepsis is also accompanied by coagulation abnormalities [37]. Thus, plasma would make it possible to study proteins involved in the coagulation cascade which would appear to be relevant for biomarker research and potential therapeutic strategy. This makes plasma a more appropriate source for the detection of nontargeted biomarkers or therapeutic targets (Table 2).

*(2) Plasma*. Proteomic analyses of plasma have been widely used to identify sepsis biomarkers. Conducted over the past 10 years, a number of them have examined plasma proteomic changes in animal models or in patients with sepsis. In 2019, a study on the plasma proteome of a mouse model of sepsis caused by five different pathogens sought to understand the molecular connections that lead to the progression of the pathology. The analysis of the different plasma samples identified a network of 84 proteins. According to bibliographic data, these proteins have already been described as being involved in human sepsis. In addition, the authors showed that these proteins could be separated into functional networks including those involved in immune suppression, vascular homeostasis, coagulation, or the complement cascade [38]. More recently, a study conducted on plasma from patients with sepsis showed an increase in acetylated truncated S100A8 and S100A9 as well as monooxidized S100A8 in nonsurviving patients with septic shock [9]. The increase in monooxidized S100A8 protein can be explained by the increased production of ROS by neutrophils and monocytes in sepsis or septic shock. This ROS production contributes to organ damage, and the protein S100A8 could also reflect an organic dysfunction. These proteins, which are in the family of alarmins, appear to be potential markers that could improve the management of patients at risk of dying of septic shock.

A major limitation to the use of plasma and serum for biomarker research is that it is not known to what extent different affected tissues alter the composition of plasma during a disease state [39]. Based on the elements developed in this paragraph, it appears that despite the limitations mentioned above, plasma is the most relevant biological sample for the identification of early sepsis biomarkers.

To conclude, mass spectrometry can analyse different types of biological samples to identify candidate sepsis biomarkers. Given the complexity of this pathology and the great heterogeneity between patients, it would seem more appropriate to analyse plasma for a protein combination whose level changes during sepsis. Such a combination would improve the sensitivity and specificity of these potential biomarkers.

The obvious disadvantage of working with human samples is that the kinetics of the pathology's evolution are not controlled and that blood sampling occurs at various stages of disease progression. Indeed, some patients will present a reduced early phase and will very quickly develop complications such as organ dysfunction associated with the pathology. Others will develop such complications later or not at all. This heterogeneity interferes with the identification of “ideal” biomarkers—in other words early, sensitive, and specific to the pathology. Therefore, many studies have focused on animal models as a first step.

## 3. Animal Models

Animal models of sepsis have been developed to reproduce the haemodynamic and molecular changes that occur in human sepsis. By studying the evolution of the pathology, these models enable us to understand the underlying mechanisms and thus identify potential biomarkers or therapeutic targets. They have the advantage of being better controlled from the viewpoint of kinetics and interindividual variability. However, the results obtained with the animal model are not always transposable to humans. These elements underline the importance of the choice of the animal model as a rationale for the identification of new biomarkers and in the search for molecules to improve the management of sepsis. The different models of sepsis clearly remain a compromise between standardisation and clinical relevance.

### 3.1. Nonsurgical Models

*(1) Injection of Exogenous Molecules*. In these models, bacterial products or endotoxins, injected intravenously (iv) or intraperitoneally (ip), replace the bacteria. They are simple to use, robust, and reproducible models. The most used molecules are lipopolysaccharides (LPS), deoxyribonucleic acid (DNA), ribonucleic acid (RNA), or synthetic oligodeoxynucleotides containing unmethylated CpG units (ODN-CpG). These patterns can be standardised by normalising the injected doses. Endotoxin-treated animals therefore present a clinical picture that is similar to sepsis with systemic arterial hypotension; impaired myocardial contractility; and increased circulating levels of lactate, tumour necrosis factor (TNF), and interleukin-6 (IL-6) [40–42]. However, the kinetics of sepsis development observed in endotoxemic shock do not mimic those observed in the patient. Indeed, it has been shown that after endotoxin injection, a strong and rapid increase in several proinflammatory cytokines was observed in mouse models in contrast to the smaller and progressive increase in sepsis patients [43]. Finally, endotoxemic models are characterized by the injection of LPS from a single bacterial strain with no initial infectious focus. LPS are mainly recognized at the extracellular stage by Toll-like receptors (TLRs) and by TLR4. The activation of these receptors will stimulate the pathways associated with inflammation, notably through the activation of the nuclear factor-*κ*B (NF-*κ*B) which regulates the transcription of numerous cytokines [44]. However, septic shock is, in most cases, caused by a polymicrobial infection that activates multiple signalling pathways. Although exogenous injection models are the most widely used according to the literature, they do not completely reproduce the characteristics of sepsis in humans (Table 3).

*(2) Injection of Bacteria*. To reduce the limitations of the models described above, models for injecting live and dead bacteria have been developed. Bacteria can be administered by intravenous, intraperitoneal, intramuscular, or intratracheal injection. The use of whole bacteria exposes the organism to numerous bacterial components that can activate different receptors in the host and contributes to the complexity of these models. These models, which are simple to set up, make it possible to generate different infectious sites. These models can be used to reproduce pneumosepsis, urosepsis, or peritonitis [45, 46]. The caecal slurry peritonitis model, used in the study of paediatric sepsis in particular, consists in injecting the caecal contents of other animals, including humans, to induce polymicrobial sepsis in the animals studied [47]. The model's advantage is that bacterial injection can be standardised by normalising the titre administered and the site and time of administration. Animals that received the bacteria presented alterations in myocardial contractility and haemodynamic and physiological changes that are associated with sepsis in humans [48]. However, these models involve the administration of high doses of bacteria to overcome host defences which effectively eliminates low doses of bacteria. These high doses will not interact with the host as in regular infection and for instance will not colonise the peritoneal cavity. Hence, this approach is not identical and will not accurately reproduce the host response which is often due to the rapid lysis of bacteria by the complement system [49]. In addition, the clinical relevance of these exogenous models could be affected by the bacterial load, the virulence of the strain of bacteria used, or the site of infection (Table 2).

### 3.2. Surgical Models

Surgical models are much more complex to set up, yet they create a more representative infectious site that better simulates a pathophysiological systemic immune response such as peritonitis.

*(1) Implantation Model*. Described in 1970, the implantation of a fibrin clot containing a standardised number of bacteria in the peritoneal cavity allows for the progressive release of the bacteria into the bloodstream (Table 3). Studies have shown that this model generates myocardial depression and a cytokine response similar to those observed in humans [51]. However, the use of a single organism in the fibrin clot is subject to the same criticisms as the injection of bacterial cultures with respect to clinical relevance (Table 3). In addition, this model requires major surgery to implant a fibrin clot in the peritoneal cavity with variable host response. The peritonitis model is therefore the currently preferred model.

*(2) Model of Peritonitis*. The peritonitis model has been widely used over the past 30 years to study the pathogenesis and therapeutic targets of sepsis [52]. This model involves the induction of intestinal lesions that cause microbial flora leakage into the normally sterile peritoneal cavity. For this purpose, caecal ligation and puncture (CLP), caecal ligation and incision (CLI), or colon ascendant stent peritonitis (CASP) can be performed [52, 53]. In all three models, the caecum, which contains a wide variety of bacteria, is perforated by one or more needle punctures (CLP), incision (CLI), or the introduction of a stent into the ascending colon distal to the ileocaecal valve (CASP). In the CLP and CLI models, the haemodynamic, metabolic, immunological, and apoptotic responses, characteristic of organ dysfunction, are more similar to those of human sepsis, which supports the validity of this model [53, 54]. The study of plasma biomarkers also indicates that CLP is clinically relevant [55]. In this model, the severity of sepsis can be modulated by the proportion of ligated caecum, their size, and the number of punctures. However, this aspect also represents a weakness of these models because the procedure is experimenter-dependent, resulting in a lack of reproducibility within and between different research groups. Furthermore, CLI is associated with remarkably high mortality. To more accurately reproduce a case of peritonitis following intestinal perforation in humans, the CASP model has been developed [50, 56]. This model is generated by the insertion of a stent, which limits blood flow without stopping it. The CASP model limits necrosis and the associated responses and generates diffuse peritonitis with a continuous bacterial translocation from the bowel to the peritoneal cavity. It leads to organ dysfunction as in the CLP and CLI models or septic patients. With this model, the severity of sepsis is adjustable according to the diameter of the stent and mortality is also associated with stent size [57]. The main limitation of these surgical models is that they are very poorly reproducible, so although they better represent the pathology, their use remains complex and limited (Table 2). In addition, the microbiota varies from one model to another and may interfere with the identification of candidate biomarkers. All of these data tend to place the CPL model as the most adequate model to model sepsis.

### 3.3. Limits Associated with the Animal Model

The sepsis models described above have been used with the animal models. To date, the mouse model remains the most widely used because it is less expensive and has a wider range of reagents available for biochemical studies compared with other species. Laboratory animals are chosen to have similar gene heritage, age, weight, and nutritional status, which does not reflect the heterogeneity among humans. Secondly, the mouse model does not have the same immune system as humans, resulting in a different form of resistance to infection than humans (Table 4). Pigs or sheep, which are more susceptible to infection, could therefore be more relevant [50]. No attempt has been made to introduce best practices, management guidelines, and standardisation in sepsis research, creating confusion with conflicting data resulting from variations in the definition of sepsis or the duration of study [58]. The animal models set up and the samples analysed as well as the time taken to collect these samples vary from one study to another. The lack of standardisation of preclinical data makes it difficult to use the results to identify potential biomarkers or therapeutic targets [59].

In this context, one could suggest withdrawing preclinical animal models. Nevertheless, it is recognized that, for instance, many of the pathways of acute inflammation have been elucidated by the rodent CLP model, considered as a pertinent polymicrobial model [60]. In addition, by refining the animal model of sepsis—i.e., by “humanising” animal diet and microbiome, by studying animals of various ages and both sexes in the presence or absence of underlying chronic comorbidities, and incorporating the basic treatment (fluids, antibiotics)—it could be possible to evaluate models of sepsis and septic shock pathological conditions closer to those of human cases with more relevance such as septic cardiomyopathy [61]. Finally, the “online” translational comparison with biological samples harvested from patients in septic shock will make it possible to confirm or overrule the relevance of a therapeutic target.

## 4. Conclusion/Discussion

The use of “secretomics”/proteomics based on MS has led to the identification of many promising biomarkers for the early diagnosis of sepsis and the prevention of organ dysfunction, particularly cardiac dysfunction. However, the validity and clinical utility of many of these biomarkers have not been tested. In clinical practice, these biomarkers must be validated to human cases of sepsis. They should then be routinely usable, in other words, they must be rapidly quantifiable and relatively cheap. Anyway, MS analyses from biological fluids appear to be more transferable to the clinical setting. Since January 2015, 1,495 studies have focused on the use of biomarkers for the diagnosis of sepsis [7]. The lack of early and specific biomarkers of sepsis and septic shock could be partly related to the fact that there are several limitations in proteomic studies that hinder the identification of clinically usable biomarkers. Indeed, although studies use proteomics to identify new biomarkers, they differ in the experimental protocol used. All of these differences result in proteomic signatures that vary from one study to another. The lack of standardisation of preclinical data makes it difficult to use the results to identify clinically relevant biomarkers. It is therefore crucial that research teams standardise their experience to provide better comparisons of results from one laboratory to another, increasing the quantity of data and limiting the heterogeneity that results from the pathology.

## Figures and Tables

**Table 1:** Nonexhaustive table of potential biomarkers recently studied.

	Biomarkers	References
Inflammation	S100A8	[9]
	High-mobility group box 1	[10]
	C-reactive protein	[11]
	Presepsin	[12]

Acute phase response	Haptoglobin	[13]
	Serum amyloid A	[14]
	Pentraxin-3	[7]

Lipid metabolism	Serum paraoxonase	[15]
	Apolipoprotein A-V	[16]

Oxidative stress	Glutathione peroxidase 3	[17]
	Histidine-rich glycoprotein	[18]

**Table 2:** Summary of the advantages and disadvantages, in the context of sepsis, for proteomic analyses of each sample source described.

Samples	Tissues/organs	Biological fluids			Exosomes
	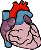 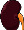	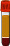	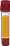	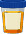	
	Kidney/heart	Plasma	Serum	Urine	
Advantages	(i) Study of the desired organ.	(i) Poorly invasive (ii) Inexpensive analyses	(i) Poorly invasive (ii) Inexpensive analyses	(i) Noninvasive	(i) Poorly invasive (ii) Provides information on the state of the cells

Disadvantages	(i) Not clinically applicable (ii) Biopsies difficult to obtain (iii) Highly invasive	(i) Wide range of concentrations	(i) Time of analysis (ii) Wide range of concentrations (iii) Loss of proteins associated with coagulation	(i) Poorly reproducible in clinics	(i) Time of analysis

**Table 3:** Summary of advantages and disadvantages of animal sepsis models.

	Model	Advantages	Disadvantages
Nonsurgical 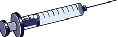	Exogenous molecules	(i) Normalisation of the injected dose and route of administration (ii) Reproduces myocardial alterations	(i) Different cytokinetics (ii) Does not reflect the complexity of human pathophysiological responses
	Bacteria	(i) Normalisation of bacteria dose (ii) Production of different infectious sites	(i) Brutal injection of bacteria (ii) Poorly reproduces the response caused by sepsis
	Caecal slurry	(i) Simple to achieve (ii) Standardisation of the injected dose and route of administration (iii) Reproducible (iv) Similar response to human sepsis	(i) Lack of hindsight on this model (ii) Model exceedingly difficult to implement
Surgical 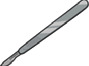	Implantation	(i) Controllable and reproducible model (ii) Progressive systemic diffusion (iii) Limited death incidence	(i) One bacterial strain used (ii) Complicated model to set up
	CLP	(i) Improved clinical relevance (ii) Severity variable according to needle diameter, number of punctures and length of ligated caecum	(i) Develops acute sepsis or intra-abdominal abscess (ii) Not controllable (iii) Dependent on experimenter (iv) Not very reproducible (v) Long to set up
	CASP	(i) Adjustable sepsis severity according to the diameter of the stent	(i) Continuous bacterial release (ii) Dependent on experimenter (iii) Long to set up
	CLI	(i) Polymicrobial model of sepsis (ii) Progressive systemic diffusion	(i) High death incidence (ii) Dependent on experimenter (iii) Poorly reproducible (iv) Long to set up

Adapted from the work of Murando et al. [50]. CLP: caecal ligation and puncture; CLI: caecal ligation and incision; CASP: colon ascendant stent peritonitis.

**Table 4:** Summary of the major differences between humans and the most used animal models of septic shock.

		Human	Mouse	Rat
		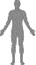	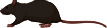	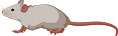
Heart rhythm 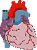		70 bpm	500-700 bpm	250-400 bpm
Respiratory rhythm 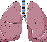		12-20/min	100-200/min	70-110/min
Surface ratio of small intestine/colon 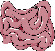		18	400	400
Endotoxin		High sensitivity	High resistance	High resistance
Immune system	Main protein in acute phase	CRP	SAP	SAP
	Largest fraction of blood cells	Neutrophils	Lymphocytes	Lymphocytes
	Complement system	High plasma activity	Poor plasma activity	Poor plasma activity

Adapted from the work of Cavaillon et al. [62]. Bpm: beats per minute; CRP: C-reactive protein; SAP: serum amyloid protein.

## Data Availability

The data supporting this review are from previously reported studies and datasets, which have been cited.
